# Laparoscopic versus open appendectomy in patients with suspected appendicitis: a systematic review of meta-analyses of randomised controlled trials

**DOI:** 10.1186/s12876-015-0277-3

**Published:** 2015-04-15

**Authors:** Thomas Jaschinski, Christoph Mosch, Michaela Eikermann, Edmund AM Neugebauer

**Affiliations:** Department for Evidence-based health services research, Institute for Research in Operative Medicine, Witten/Herdecke University, Ostmerheimer Str. 200 (building 38), 51109 Cologne, Germany

**Keywords:** Systematic review, Overview, Appendicitis, Appendectomy, Laparoscopy

## Abstract

**Background:**

Several systematic reviews (SRs) of randomised controlled trials (RCTs) comparing laparoscopic versus open appendectomy have been published, but there has been no overview of SRs of these two interventions. This overview (review of review) aims to summarise the results of such SRs in order to provide the most up to date evidence, and to highlight discordant results.

**Methods:**

Medline, Embase, Cinahl, the Cochrane Database of Systematic Reviews and the Database of Abstracts of Reviews of Effects were searched for SRs published up to August 2014. Study selection and quality assessment using the AMSTAR tool were carried out independently by two reviewers. We used standardised forms to extract data that were analysed descriptively.

**Results:**

Nine SRs met the inclusion criteria. All were of moderate to high quality. The number of randomized controlled trials (RCTs) they included ranged from eight to 67. The duration of surgery pooled by eight reviews was 7.6 to 18.3 minutes shorter using the open approach. Pain scores on the first postoperative day were lower after laparoscopic appendectomy in two out of three reviews. The risk of abdominal abscesses was higher for laparoscopic surgery in half of six meta-analyses. The occurrence of wound infections pooled by all reviews was lower after laparoscopic appendectomy. One review showed no difference in mortality. The laparoscopic approach shortened hospital stay from 0.16 to 1.13 days in seven out of eight meta-analyses, though the strength of the evidence was affected by strong heterogeneity.

**Conclusion:**

Laparoscopic and open appendectomy are both safe and effective procedures for the treatment of acute appendicitis. This overview shows discordant results with respect to the magnitude of the effect but not to the direction of the effect. The evidence from this overview may prove useful for the development of clinical guidelines and protocols.

**Electronic supplementary material:**

The online version of this article (doi:10.1186/s12876-015-0277-3) contains supplementary material, which is available to authorized users.

## Background

Appendicitis is the most common reason for acute abdominal pain with a lifetime risk of 8.6% for males and 6.7% for females [1]. The treatment of choice is the surgical removal of the inflamed appendix by using open appendectomy (OA) first described by McBurney in 1894 or by using laparoscopic appendectomy (LA) specified by Semm in 1983 [2,3]. Both surgical methods are safe and well established in clinical practice but there has been a controversy about which surgical procedure is the most appropriate. Therefore, several systematic reviews (SRs) have been conducted summarising, assessing and synthesising the data from primary studies. However, despite similar research questions and methodology, SRs show discordant results for individual endpoints.

The Cochrane Collaboration has introduced a new type of review called an overview of SRs. This method offers a new approach for synthesising the results of the increasing number of SRs. An overview summarises, evaluates and compiles the available evidence from SRs relevant to a single health problem [4]. However, only a few methodological publications on how to conduct an overview are available [4,5]. Therefore, due to a lack of methodological and reporting standards, overviews have varied substantially in performance and in methodological quality, and the benefit of overviews has not been clearly established [6,7]. The purpose of this paper is to conduct an overview of SRs that compares LA versus OA to provide the most up to date evidence and to analyse the reasons for discordant results.

## Methods

### Systematic literature search

Medline, Embase, Cinahl, the Cochrane Database of Systematic Reviews (CDSR) and the Database of Abstracts of Reviews of Effects (DARE) were searched for SRs that compared LA versus OA in patients with suspected appendicitis by using a combination of text words and database specific controlled vocabulary without any restrictions regarding publication date or language (see Additional file 1 available online).The last update search was conducted on August 27, 2014. To identify additional citations missed by electronic searches, references of included studies were checked manually. There is no review protocol or registration available.

### Study selection

Two authors independently screened search results by title and abstract to identify potentially relevant SRs according to inclusion criteria created a priori. We included only the most recent version of a SR when updated versions were available. SRs without any systematic search in at least one database or without critical appraisal of included RCTs were excluded. After the retrieval of potentially relevant studies, full texts were checked against the inclusion criteria once again. Any disagreement was resolved by consensus. In the case of unresolvable discrepancies, a third reviewer was involved in the discussion.

### Data extraction and quality assessment

The standardised data extraction form summarised year of publication, inclusion criteria, databases searched, search period and the number of included RCTs. We categorised the relevant outcomes as primary or secondary. Primary outcomes were pain on postoperative day 1, wound infections, intra-abdominal abscesses and mortality. Secondary outcomes were duration of surgery, conversions, length of stay, in-hospital costs (including surgery costs) and time until return to work. We extracted pooled effect sizes and corresponding confidence intervals if reported. To analyse the overlap of included SRs, we used a citation matrix that crosslinks the SRs with their included RCTs to compute the “covered area” (CA) and the “corrected covered area” (CCA) according to Pieper [8]. For the evaluation of the methodological quality of the included SRs, we applied the eleven-item AMSTAR tool due to its reliability, construct validity and feasibility [9-11]. Each assessment question was rated with “yes”, “no”, or “can’t answer”. The data extraction, citation matrix and assessment of methodological quality were conducted by one author and checked by a second. Any disagreements were resolved by discussion or by consultation with a third reviewer.

## Results

### Study selection process

**Figure 1:**
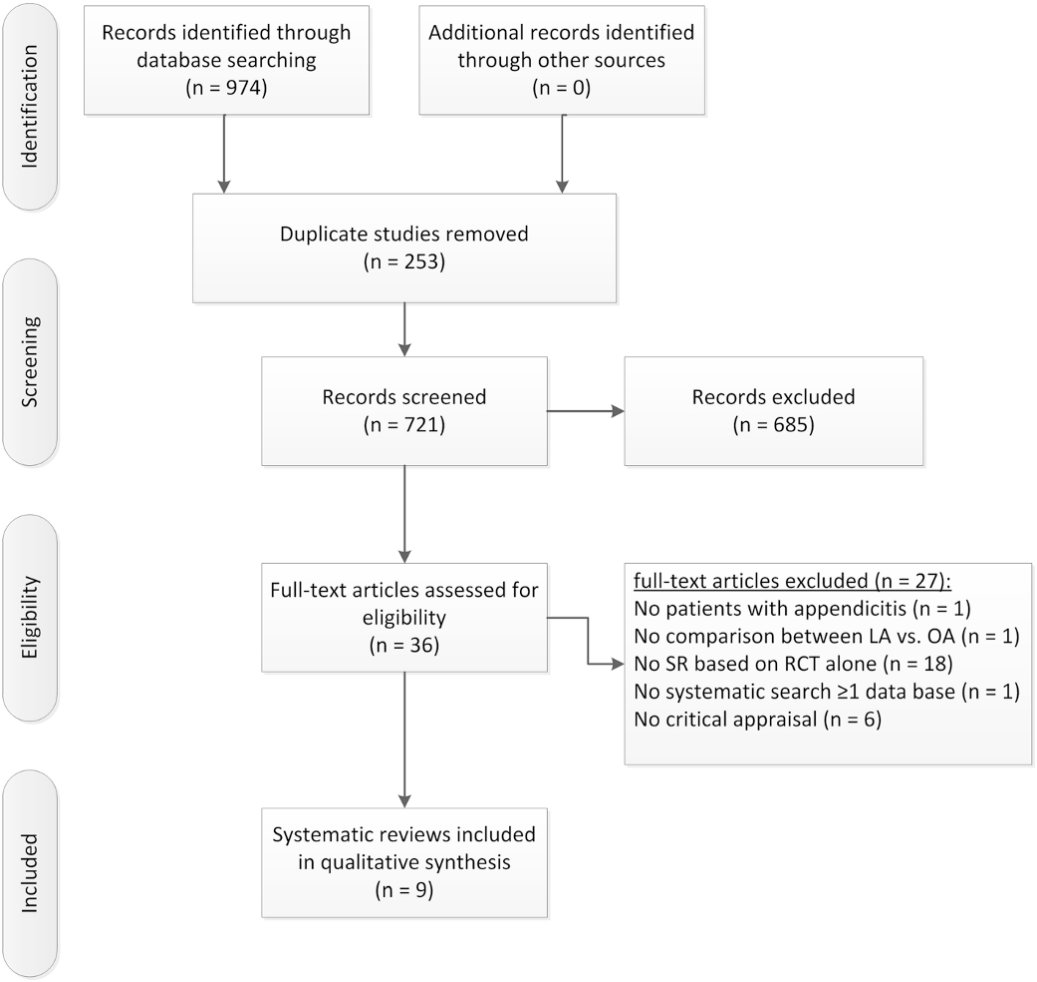
Flow chart of study selection.

The study selection process is presented in Figure 1. A total of 974 records were identified through the systematic search. After removing the duplicates, the title and abstract of 721 references were screened for meeting the inclusion criteria. After the retrieval of 36 potentially relevant full-text articles (including relevant supplements or appendices), 27 were excluded for the following reasons. One SR did not address patients with suspected appendicitis, one analysed LA without comparison, 18 included study designs of both RCTs and non-RCTs, one did not search systematically in at least one electronic database, and six had no quality assessment of the RCTs. Thus, a total of nine SRs were included in this overview [12-20].

### Description of systematic reviews

All the included SRs published in English between 1998 and 2012 performed meta-analyses. Their characteristics are shown in Table 1. The included SRs analysed between eight and 67 RCTs on LA versus OA in patients with suspected appendicitis. Two of the SRs included only RCTs that had recruited only adults. Seven of the SRs applied a language limitation. The number of searched databases ranged from one to six. Medline was the most frequently searched electronic database followed by Cochrane Library and Embase. The number of analysed outcome measures ranged from one to 22. The methodological quality of the included RCTs was assessed by using the Jadad scale [14,15,17], the modified Jadad scale [20], the 10-point scale proposed by Solomon [13,19], the McMaster University method [16], the Cochrane risk of bias tool [18] and in one SR, the authors used their own checklist [12].

**Table 1:** **Study characteristics**

Study	Inclusion criteria	Data bases, (search period), number of included studies
**Golub 1998** [13]	Comparison between LA and OA in adults formally, RCT, published in English	Medline (09/1992 – 07/1997), 16 studies included
**Meynaud-Kraemer 1999** [16]	Comparison between LA and OA in adults, RCT, published in English/ French/ German	Medline (search period not reported), 8 studies included
**Temple 1999** [19]	Patients with preoperative diagnosis of acute appendicitis comparison between LA and OA, RCT (random allocation of patients into LA or OA), published in English, LA intended to be therapeutic rather than diagnostic, ≥ 80% of randomized patients were followed up for at least one of the main outcomes	Medline (01/1990 - 03/1997), 12 studies included
**Bennett 2007** [12]	Comparison between LA and OA for acute appendicitis, RCT, allocation concealment, published in English peer-reviewed journal, not as abstract reported	Cochrane Library, Medline, Embase, (1995 – 05/2006), 33 studies included
**Li 2010** [14]	Comparison between LA and OA, RCT, published in English, desirable outcome reported	Medline, Embase, Central (01/1990-12/2009), 44 studies included
**Liu 2010** [15]	Comparison between LA and OA, ≥9 years old, not pregnant, no drug abuse, no psychiatric disorder, RCT, no language restriction	Medline, Embase, Cochrane Library (01/1992 – 01/2008), 16 studies included
**Sauerland 2010** [18]	Comparison between LA and OA in adults or children with symptoms and signs of acute appendicitis, RCT, adequate concealment of allocations, no language restriction, authors of an abstract were requested for details and had to provide full information on their trial, more than 50% appendix specimens without histological signs of inflammation	Medline, Embase, Cochrane Library, Lilacs, CNKI, SciSearch Cochrane/Medline (- 15/04/2010) remaining databases (-08/2009), 67 studies included
**Wei 2011** [20]	Comparison between LA and OA, RCT, published in English, most recent article from the same institution when several studies reporting the same patients, at least four variables of interest could derived from the published results, no variations on the standard laparoscopic technique including laparoscope-assisted or single-trocar appendectomy	Medline, Embase, Current Contents (01/1992 – 02/2010), 25 studies included
**Ohtani 2012** [17]	Comparison between LA and OA, RCT, abstracts excluded (only included if full text was published), published in English, at least one of the outcome measures mentioned	Medline, Embase, Central, Science Citation Index (01/1990 – 02/2012), 39 studies included

### AMSTAR ratings for the reviews

The AMSTAR ratings are summarised in Table 2. All the included SRs were of moderate or high quality. Lack of an assessment of publication bias and the absence of a statement of potential sources of support were the most common flaws. Three SRs published before 2000 failed to conduct a comprehensive literature search by using only one database. One SR did not report the search period [16]. Indeed, key words and MESH terms were stated frequently, but no author provided the complete search strategy. In their analysis of the RCTs, all the SRs presented the study characteristics, performed a critical appraisal, and used adequate methods for combining the results.

**Table 2:** **AMSTAR ratings**

AMSTAR criteria	Golub 1998	Meynaud-Kraemer 1999	Temple 1999	Benett 2007	Li 2010	Liu 2010	Sauerland 2010	Wei 2011	Ohtani 2012
**A priori design**	**o**	**o**	**+**	**o**	**o**	**o**	**+**	**o**	**o**
**Duplicate study selection and extraction**	**+**	**-**	**+**	**+**	**+**	**+**	**+**	**o**	**o**
**Literature search comprehensive**	**-**	**-**	**-**	**+**	**+**	**+**	**+**	**+**	**+**
**Status of publication used as criteria**	**+**	**+**	**+**	**+**	**+**	**+**	**+**	**+**	**+**
**Excluded/included list provided**	**-**	**+**	**+**	**+**	**+**	**-**	**+**	**-**	**-**
**Study characteristics provided**	**+**	**+**	**-**	**+**	**+**	**+**	**+**	**+**	**+**
**Quality assessed/presented**	**+**	**+**	**-**	**+**	**+**	**+**	**+**	**+**	**+**
**Quality impacted conclusions**	**+**	**+**	**+**	**+**	**+**	**+**	**+**	**+**	**+**
**Heterogeneity tested before combining**	**+**	**+**	**+**	**+**	**+**	**+**	**+**	**+**	**+**
**Publication bias assessed**	**o**	**-**	**-**	**-**	**+**	**+**	**+**	**+**	**-**
**Conflict stated**	**-**	**-**	**-**	**-**	**+**	**-**	**+**	**+**	**-**

“+” = yes (clearly done); “-“= no (clearly not done); “o” = can’t answer.

### Overview of primary and secondary outcomes

The results of the extracted primary and secondary outcomes are given in Table 3. Three SRs showed a reduction of pain on postoperative day 1 in favour of LA compared with OA, but the effect sizes (which varied from -0.8 to -0.7 points on a 10 points VAS) were significant in only two of the SRs. These findings were affected by strong heterogeneity among the primary studies. The incidence of wound infections was significantly less for LA and the odds ratio (OR) ranged from 0.3 to 0.52 with low heterogeneity across the RCTs. Six SRs computed the OR for intra-abdominal abscesses; the values ranged from 1.56 to 2.29. Three meta-analyses showed no significant difference between LA and OA but three others detected significantly higher rates of intra-abdominal abscesses for LA. Only one SR analysed mortality rates; based on seven RCTs, this SR found that the mortality rates were not significantly different between the two surgical approaches.

**Table 3:** **Primary and secondary outcomes**

	Golub 1998	Meynaud-Kraemer 1999	Temple 1999	Bennett 2007	Li 2010	Liu 2010	Sauerland 2010	Wei 2011	Ohtani 2012
**Primary outcomes**									
pain on postoperative day 1 [WMD, 95%-CI]	-	-	-	LA (5) [-0.8, -1.84;0.25]	LA* (8) [-0,7, -1.22;-0.19]	-	LA* (15) [-0.82, -1.14;-0.49]	-	-
wound infections [OR, 95%-CI]	LA* (16) [0.3, 0.19;0.47]†	LA* (8) [0.33, 0.18;0.61]†	LA* (10) [0.4, 0.24;0.69]†	LA* (25) [0.52, 0.39;0.70]	LA* (31) [0.45, 0.34;0.59]†	LA* (13) [0.51, 0.36;0.73]†	LA* (50) [0.43, 0.34;0.54]†	LA* (20) [-, 0.3;0.56]†	LA* (32) [0.46, 0.34;0.62]†
intraabdominal abscesses [OR, 95%-CI]	OA (15) [2.2, 0.88;6.64]†	-	OA (6) [1.94, 0.68;5.58]†	OA* (25) [2.29, 1.48;3.53]†	OA* (17) [1.56, 1.01;2.43]†	-	OA* (45) [1.87, 1.19;2.93]†	OA (12) [-, 0.93;2.14]†	-
mortality	-	-	-	-	-	LA (7)	-	-	-
[OR, 95%-CI]						[0.97, 0.29;3.25]†			
**secondary outcomes**									
duration of surgery [WMD, 95%-CI]	OA* (16) [18.3, -;-]	-	OA* (8) [18.1, 12.87;23.15]	OA* (22) [14.61, 9.04;20.19]	OA* (36) [12.35, 7.99;16.72]	OA* (8) [7.6, 6.03;9.17]	OA* (38) [10,24 5.51;14.97]	OA* (25) [10.71, 6.76;14.66]	OA* (36) [13.12, 9.72;16.51]
overall conversion rate {range}	9.7% (14) {0%-20%}	-	11% (-) {5%-20%}	-	-	-	-	-	-
length of hospital stay [WMD, 95%-CI]	LA* (14) [-0,61, -;-]	-	LA (8) [-0.16, -0.44;0.15]†	LA* (18) [-0.62, -1.05;-0.18]	LA* (32) [-0.6, -0.85;-0.36]	LA* (8) [-0.82, -0.93;-0.7]	LA* (34) [-1.13, -1.51;-0.74]	LA* (23) [-0.68, -1.02;-0.35]	LA* (33) [-0.79, -1.06;-0.52]
in-hospital costs (including surgery costs) [OR, 95%-CI]	-	-	-	-	-	-	OA* (6) [1.32, 0.42;2.22]	-	OA (7) [-]
time until return to work [WMD, 95%-CI]	-	-	-	-	-	-	LA (8) [-1.6, -5.22;2.02]	LA* (11) [-3.09, -5.22;-0.97]	LA* (10) [-3.18, -5.09;-1.27]

**LA**, in favour for laparoscopic appendectomy; **(n)**, number of studies included for analysis; **OA**, in favour for open appendectomy; **OR**, Odds ratio; **WMD**, weighted mean differences; **-**, not reported; *direction of effect statistically significant (p ≤ 0.05); **†** homogeneous effect size.

Based on data pooled from eight SRs, the duration of surgery by LA took 7.6 to 18.3 minutes longer than by OA, though the results were limited by high heterogeneity. Two SRs determined the overall conversion rate to be 9.7% and 11%, with values ranging from 5% to 20% and from 0% to 20%, respectively. LA compared with OA led to a reduction in length of hospital stay of 0.16 to 1.13 days. These findings were significant in seven of eight SRs, though limited by high heterogeneity. The in-hospital costs, including surgery costs, were higher for the laparoscopic approach. For recovery time, the results of three SRs showed a trend in favour of the laparoscopic approach, but only two meta-analyses showed a significant reduction of three days in time until return to work.

### Citation matrix

Table 4 shows the citation matrix crosslinking nine SRs with 81 primary studies sorted by publication date in ascending order. Using Pieper’s method, the covered area (CA) and the corrected covered area (CCA) was 35.7% and 24.6%, demonstrating a very high degree of overlap.

**Table 4:** **Citation matrix**

Systematic review included RCTs	Golub 1998	Meynaud-Kraemer 1999	Temple 1999	Bennett 2007	Li 2010	Liu 2010	Sauerland 2010	Wei 2011	Ohtani 2012
	N = 16	N = 8	N = 12	N = 33	N = 44	N = 16	N = 67	N = 25	N = 39
DeWilde 1991							x		
Attwood 1992	x	x	x		x		x		x
Kum 1993	x	x	e^FU^		x		x	x	x
Olsen 1993							x		
Tate 1993	x	x	x		x		x	x	x
Hebebrand 1994		x					x		
Eichen 1994							x		
Frazee 1994	x	x	x		x	x	x	x	x
Jadallah 1994							x		
Rohr 1994	x		e^FT^				e^AR^		
Martin 1995	x	x	x	x	x	x	x	x	x
Ortega 1995	x	x	x	x	x	x	x		x
Settmacher 1995							x		
Cox 1996	x		x	x	x	x	x	x	x
Hansen 1996	x		x	x	x	x	x	x	x
Hart 1996	x		x	x	x		x		x
Lejus 1996				x	x		x		x
Mutter 1996	x		x	x	x		x	x	x
Williams 1996	x		x	x	x	x	x	x	x
Pozo 1996							x		
Yin 1996					x		x		
Kazemier 1997	x		x	x	x		x	x	x
Laine 1997	x		x	x	x		x		
Macarulla 1997				x	x	x	x	x	x
Minné 1997	x			x	x		x	x	x
Reiertsen 1997	x		e^FU^	x	x		x		x
Schippers 1997							x		
Sezeur 1997							x		
Yeung 1997							x		
Heikkinen 1998				x	x		x	x	x
Stare 1998							x		
Sun 1998							x		
Zhang 1998				e^FT^			x		
Klingler 1998					x		x	x	x
Witten 1998							x		
Barth 1999		x^US^					x		
Bauwens 1999							x		
Hellberg 1999				x	x	x	x	x	x
Hellberg 2001					e^ra^		x		
Enochsson 2001				e^SA^	e^ra^		x		
Kald 1999				x	x		x		
Perner 1999							x		
Özmen 1999				x	x		x		x
Navarra 2000							x		
Nordentoft 2000				x	x		x		
Huang 2001				x	x	x	x		x
Helmy 2001				x	x		x		x
Larsson 2001				e^DL^	e^DL^		x		
Lavonius 2001				x	x		x		
Long 2001				x	x	x	x	x	x
Pedersen 2001				x	x	x	x	x	x
Al-Mulhim 2002				x	x		x		x
Little 2002					x		x		
Bruwer 2003				x	x		x		x
Karadayi 2003				x	x		x		
Milewczyk 2003				x	x		e^AC^	x	x
Vallribera 2003							x		
van Dalen 2003				e^DL^	e^DL^		x		
Oka 2004				x			e^NR^	x	x
Ignacio 2004				x	x	x	x	x	x
Lintula 2001					e^ra^		x	x	x
Lintula 2002					e^ra^		x		
Lintula 2004				x	x		x		
Katkhouda 2005				x	x	x	x	x	x
Olmi 2005				x		x		x	x
Moberg 2005				x	x	x	x	x	x
Kaiser 2006							x		
Ricca 2007					x		x		
Tzovaras 2007					x		x	x	
Bolla 2008							x		
Moirangthem 2008					x		x		
Kehagias 2008						x			
Kaplan 2009					x		x		
Kehagias 2009							x		
Simon 2009					x		x		
Wei 2010					x		x	x	x
Tzovaras 2010									x
Kouhia 2010									x
Shirazi 2010									x
Khalil 2011									x
Clarke 2011									x

x: included in review; x^US^: unpublished study included since trial was known to the authors; e^AC^: no allocation concealment; e^AR^: only as abstract reported and author request remained unanswered; e^FU^: follow-up < 80%; e^FT^: full text not available; e^NR^: not randomised; e^DL^: use of diagnostic laparoscopy followed by open appendicectomy in the laparoscopy arm; e^SA^: analysed a subgroup of the patients reported in a previous paper; e^ra^: not the most recent or highest quality article.

Despite having the same research question and overlapping search periods, the SRs did not include the same set of RCTs due to their different exclusion criteria. In one case, the authors had no access to the full text [19], in another the author of an abstract did not answer the request for further information [18]. Differences in the study selection process also resulted from different inclusion criteria. For example, the authors of one SR excluded two studies due to low follow-up [19]. Additionally, there was discordance in excluding studies for the same inclusion criteria. Three SRs analysed a trial in which the assignment of patients to the intervention group had not been random but had instead been based on the schedule of the attending surgeon on call. Meynaud-Kraemer et al. [16] included one RCT which had been published after their initial literature search because one author providing the needed data was also involved in the primary study [16]. A further comparison of included and excluded studies was not possible since the references of excluded studies were reported only in five SRs [12,18,19,14,16].

## Discussion

This overview aims to summarise SRs comparing LA versus OA for patients with suspected appendicitis to provide the most up to date evidence, and to highlight discordant results. Nine relevant SRs meeting all the inclusion criteria could be identified. Although we imposed no language restriction in order to prevent publication bias, the only relevant SRs we found were published in English. Our overview shows that LA and OA have been extensively analysed by RCTs and SRs, and that both approaches are safe and effective techniques for the treatment of suspected appendicitis and are associated with good clinical outcomes and little harm. The trend for reduced pain on postoperative day 1 after LA was lower in two out of three SRs but limited by high heterogeneity. The risk of abdominal abscesses was higher following LA in three out of six meta-analyses. The most clear and consistent finding with low heterogeneity was the reduction of wound infections after LA. The results of seven pooled RCTs showed no difference in mortality. The laparoscopic approach shortened hospital stay in eight meta-analyses, but again the data was heterogeneous.

The quality of the included SRs was moderate to high and thus met our quality evaluation criterion. Due to poor reporting, we could often not answer the AMSTAR item about ‘a priori design’ using only the publication for the assessment and not making any enquiries to the authors.

Not requesting further information from the authors in cases where data was missing is one weakness of our overview. For instance, there is a loss of information because the data on pain was not extracted from one study because it did not report the moment of pain measurement [13]. Because we extracted only outcomes determined a priori, our presentation of the endpoints is incomplete. To reduce the risk of bias in our work, we included only those SRs for which a search in at least one electronic database had been conducted and which assessed the included RCTs critically by using a checklist.

Despite the different publication dates and number of included RCTs, the direction of effects for the analysed endpoints was the same and did not change over time. The direction of effect size estimates for wound infections and for the duration of surgery was significant in all SRs; however, there was a high variation in these effect size estimates. The discordant results are probably based on a combination of methodological causes and content-related reasons. Although the SRs had the same research question, they included different studies because they used different databases, search strategies and search periods. One SR did not specify the search strategy at all [16] and eight provided only keywords and general terms [12-15,17-20]. Thus, not a single search is completely comprehensible. Additional sources of discordant results are the different criteria used to select studies for inclusion. Some authors excluded studies due to a low follow-up, the lack of full text, insufficient resources to obtain the relevant paper, or language restrictions. In this overview, there is a low risk of bias concerning the study selection and the data extraction process since almost all the SRs conducted these quality assurance steps. Moreover, for pooling the data, the authors of the SRs applied either the fixed effect model or the random effect model or a combination of both depending on the heterogeneity. The authors of only three SRs contacted the authors of the primary studies to obtain missing data. One author request revealed that a trial classified as a randomised study used an inadequate sequence generation by allocating the patients according to the schedule of the attending surgeon on call.

Our research question focused on only two treatment procedures, but for decision makers, clinicians and patients, an overview including further types of interventions for appendectomy would be more interesting for their daily work and decision-making.

One fundamental disadvantage of overviews is the delayed integration of results from available primary studies. Overviews cannot reflect all the current evidence. In our example, the last published SR conducted its search in February 2012, so that RCTs published after this date have not been considered here. Thus, there is a lack of evidence of more than two years in our work. However, the direction of the effect size estimates is consistent for the analysed outcomes among the SRs and did not change over time. This makes it unlikely that the results of more recent RCTs would change the confidence in the effect estimates. The strength of SRs is their pooling of data on a particular problem from multiple RCTs. In an overview, it is not possible to adopt the methods for pooling data that are used in a SR without special modification, but even if the methods were so modified, success would be only partial because of the poor quality of reporting. Consequently, the results of this overview are presented only descriptively [6].

## Conclusion

The comparison between LA and OA has been intensively analysed in over 70 RCTs; and further studies would unlikely change the results of SRs. Thus researchers and sponsors should rather focus on assessing new surgical approaches comparing single incision LA versus conventional three port LA for which there is currently insufficient evidence [21,22]. Indeed, the surgical appendectomy remains the standard treatment; however, conservative antibiotic therapy of acute appendicitis might be used in selected cases or in conditions where surgical approaches are contraindicated [23,24].

LA and OA are safe and effective procedures for the treatment of acute appendicitis in clinical practice. The direction of the pooled effects was consistent among the SRs. The evidence from this overview could be used for the development and updating of guidelines and protocols [25].

## Additional file

Additional file 1:
**Search strategies.**

## Abbreviations

CA: Covered area
CCA: Corrected covered area
LA: Laparoscopic appendectomy
OA: Open appendectomy
RCT: Randomised controlled trial
SR: Systematic review
