# Supplementary material for: Laparoscopic versus open appendectomy in patients with suspected appendicitis: a systematic review of meta-analyses of randomised controlled trials
Source: BMC Gastroenterol. 2015 Apr 15;15:48. doi: 10.1186/s12876-015-0277-3 (PMC4399217; doi:10.1186/s12876-015-0277-3)
Supplement: Additional file 1: — Search strategies. [file 12876_2015_277_MOESM1_ESM.pdf]

S1: Search strategy for Embase (database) via Embase (search engine)

((‘meta-analysis’/exp OR ‘systematic review’/exp or (meta NEXT/1 analys*):ab,ti or metaanalys*:ab,ti OR (systematic NEXT/1 (review* OR overview*)):ab,ti) OR ((Cancerlit:ab OR cochrane:ab OR embase:ab OR psychlit:ab OR psyclit:ab OR psychinfo:ab OR psycinfo:ab OR cinahl:ab OR cinhal:ab OR ‘science citation index’:ab OR bids:ab)) OR ((‘reference lists’:ab OR bibliograph*:ab OR (hand NEXT/1 search*):ab OR (manual NEXT/1 search*):ab OR ‘relevant journals’:ab)) OR ((‘data extraction’:ab OR ‘selection criteria’:ab) AND review:it))
AND
('appendectomy'/exp OR 'appendicitis'/exp OR 'acute appendicitis'/exp OR (appendic* OR appendec* OR appendek* OR apendic* OR apendec* OR apendek*):ab,ti**)**

S2: Search strategy for Medline (database) via Pubmed (search engine)

((systematic review [ti] OR meta-analysis [pt] OR meta-analysis [ti] OR systematic literature review [ti] OR (systematic review [tiab] AND review [pt]) OR consensus development conference [pt] OR practice guideline [pt] OR cochrane database syst rev [ta] OR acp journal club [ta] OR health technol assess [ta] OR evid rep technol assess summ [ta])
OR ((evidence based[ti] OR evidence-based medicine [mh] OR best practice* [ti] OR evidence synthesis [tiab])
AND
(review [pt] OR diseases category[mh] OR behavior and behavior mechanisms [mh] OR therapeutics [mh] OR evaluation studies[pt] OR validation studies[pt] OR guideline [pt]))
OR ((systematic [tw] OR systematically [tw] OR critical [tiab] OR (study selection [tw]) OR (predetermined [tw] OR inclusion [tw] AND criteri* [tw]) OR exclusion criteri* [tw] OR main outcome measures [tw] OR standard of care [tw] OR standards of care [tw])
AND
(survey [tiab] OR surveys [tiab] OR overview* [tw] OR review [tiab] OR reviews [tiab] OR search* [tw] OR handsearch [tw] OR analysis [tiab] OR critique [tiab] OR appraisal [tw] OR (reduction [tw]AND (risk [mh] OR risk [tw]) AND (death OR recurrence)))
AND
(literature [tiab] OR articles [tiab] OR publications [tiab] OR publication [tiab] OR bibliography [tiab] OR bibliographies [tiab] OR published [tiab] OR unpublished [tw] OR citation [tw] OR citations [tw] OR database [tiab] OR internet [tiab] OR textbooks [tiab] OR references [tw] OR scales [tw] OR papers [tw] OR datasets [tw] OR trials [tiab] OR meta-analy* [tw] OR (clinical [tiab] AND studies [tiab]) OR treatment outcome [mh] OR treatment outcome [tw]))
NOT
(letter [pt] OR newspaper article [pt] OR comment [pt])**)**
AND
("Appendectomy"[Mesh] OR "Appendicitis"[Mesh] OR appendic*[TIAB] OR appendec*[TIAB] OR appendek*[TIAB] OR apendic*[TIAB] OR apendec*[TIAB] OR apendek*[TIAB])

S3: Search strategy for Cochrane Database of Systematic Reviews (CDSR) and Database of Abstracts of Reviews of Effects (DARE) (database) via Cochrane Library (search engine)

Mesh descriptor: [Appendectomy] explode all trees OR Mesh descriptor: [Appendicitis] explode all trees OR (appendic* OR appendec* OR appendek* OR apendic* OR apendec* OR apendek*):ti,ab,kw

S4: Search strategy for CINAHL (database) via EBSCOhost (search engine)

(((MH "Meta Analysis") OR (MH "Systematic Review") OR (TX "meta analys*") OR (TX metaanalys*) OR (TX systematic N1 (review* OR overview*)) OR AB cancerlit OR AB cochrane OR AB embase OR AB psychlit OR AB psyclit OR AB psychinfo OR AB psycinfo OR AB cinahl OR AB cinhal OR AB "science citation index" OR AB bids OR AB "reference lists" OR AB bibliograph* OR AB "hand search*" OR AB "manual search*" OR AB "relevant journals" OR ((AB "data extraction" OR AB "selection criteria") AND PT review)) NOT (PT letter OR PT editorial OR (MH "Animals+") NOT ((MH "Animals+") AND (MH "Human")))) AND ((MH "Appendectomy") OR (MH "Appendicitis") OR TX appendic* OR TX appendec* OR TX appendek* OR TX apendic* OR TX apendec* OR TX apendek*)
